# Comparison of the Clinical Efficacy of Salbutamol with Jet and Mesh Nebulizers in Asthmatic Children

**DOI:** 10.1155/2018/1648652

**Published:** 2018-03-13

**Authors:** Norihide Murayama, Kikuno Murayama

**Affiliations:** MURAYAMA Pediatrics, 3-2-33 Nagayoshi-Nagahara-Higashi, Hirano-ku, Osaka-shi, Osaka 547-0013, Japan

## Abstract

**Background:**

Ultrasonic, jet, and mesh nebulizers have all been used in the treatment for asthma. Mesh nebulizers reportedly offer the best inhalation efficiency.

**Methods:**

This study aimed to clarify the utility of the mesh nebulizer, compared to jet nebulizers, in the treatment of pediatric asthma patients. Participants included 88 children <6 years old who were receiving treatment for asthma at Murayama Pediatric Clinic. Heart rate, peripheral oxygen saturation in arterial blood, and Mitsui symptom scores were compared before and after treatment with a mesh nebulizer (*n* = 43) or jet nebulizer (*n* = 45) using a salbutamol inhalation solution (0.2 ml for children ≧ 2 years old, *n* = 51; 0.1 ml for children < 2 years old, *n* = 37).

**Results:**

Other than required inhalation time, clinical findings did not differ between mesh and jet groups. In both groups, heart rate increased significantly in patients treated with 0.2 ml (1000 microg) of salbutamol.

**Conclusions and Clinical Relevance:**

The required inhalation time of the mesh nebulizer was superior to the jet nebulizer. Children ≧ 2 years with mild asthma attacks experienced a significantly increased heart rate in both groups. The dose of salbutamol (0.2 ml for ≧2 years) used for asthma attacks should be reconsidered in mild asthma.

## 1. Introduction

Hospitals and clinics in Japan have used bronchodilators for many years in the treatment of acute asthmatic attacks. Nebulized bronchodilators have many advantages compared to oral medication due to direct delivery to the obstructed airway sites. Inhalation therapy for asthma is currently broadly divided into the following three methods: nebulizer, pressurized metered-dose inhaler (pMDI), or dry powder inhaler. Electronic nebulizers are further categorized as ultrasonic, jet, and mesh varieties, based on the three methods of aerosol particle generation. The advantages of mesh nebulizers include more uniform particle generation, lower power consumption, and lower noise levels [1]. A uniform particle size with narrow particle diameter distribution allows more of the drug to reach sites of airway obstruction, theoretically providing better clinical efficacy. We hypothesized that mesh nebulizers were the best method to deliver medication to obstructed airways during status asthmatics.

This study compared the clinical utility of the eMotion mesh nebulizer (PARI, Starnberg, Germany) and a conventional jet nebulizer (Turbo Boy-N; PARI).

## 2. Methods

In the Murayama Pediatrics Clinic, Japanese guidelines suggest oral best 2 agonists for mild asthma. Acute attacks should be treated with simultaneous leukotriene agonist and beta 2 agonist inhalation. Leukotriene oral administration and steroid inhalation are recommended for long term control [2].

This study included 88 patients < 6 years of age who were treated for mild asthma attacks at Maruyama Pediatric Clinic between October 2003 and January 2004 and were unable to perform respiratory function testing (spirometry). After informed consent, patients were randomly assigned to receive treatment with either a mesh- or jet-type electronic nebulizer. For children <2 years old (*n* = 37), a total volume of 1 ml was used, comprising 0.1 ml of salbutamol inhalation solution and 0.9 ml of saline. For children ≧ 2 years old (*n* = 51), the total volume was 1 ml, comprising 0.2 ml of salbutamol inhalation solution and 0.8 ml of saline. Treatment was administered by continuous inhalation via a mask. The required inhalation time was measured using a stopwatch. Peripheral oxygen saturation (SpO2) and heart rate were measured using a pulse oximeter (N-65; Nellcor, Minneapolis, MN) before and after 10 min of inhalation therapy. Clinical symptom scores were also evaluated before and after inhalation therapy. All clinical symptom scores were evaluated by two physicians. Asthma attack symptoms were assessed using Mitsui's symptom score [3], which is used in pediatrics. This score includes dyspnea (0, none; 1, chest retractions; 2, orthopnea), rhonchi (0, none; 1, mild; 2, severe; 3, attenuated), wheezing (0, none; 1, positive; 2, attenuated), cyanosis (0, absent; 1, present), difficulty with conversation (0, absent; 1, present), and clouding or loss of consciousness (0, absent; 1, present). The highest possible total score is 10, with scores 1–3, 4–6, and 7–10 considered to represent mild, moderate, and severe attacks, respectively.

Statistics used JSTAT for Windows version 17.1 software (Dr. Masato Satoh, Tokyo, Japan). Comparisons between continuous variables used an independent two-tailed *t*-test. Comparisons of symptom scores before and after treatment used a paired two-tailed *t*-test. All tests were considered significant at *p* < 0.05.

## 3. Results

Mean age, heart rate, SpO2, and clinical symptom scores did not significantly differ between jet and mesh nebulizer groups before and after inhalation therapy

### 3.1. Inhalation Time

Mean inhalation times were shorter in the mesh group (236 ± 22.5 seconds) than in the jet group (364 ± 53.9 s) for patients < 2 years old (*p* < 0.001, Table 1). Mean inhalation times also were shorter in the mesh group (248 ± 31.4 s) than in the jet group (377 ± 58.7 s) for patients ≧ 2 years old (*p* < 0.001, Table 2).

### 3.2. Heart Rate before and after Inhalation

**Figure 1:**
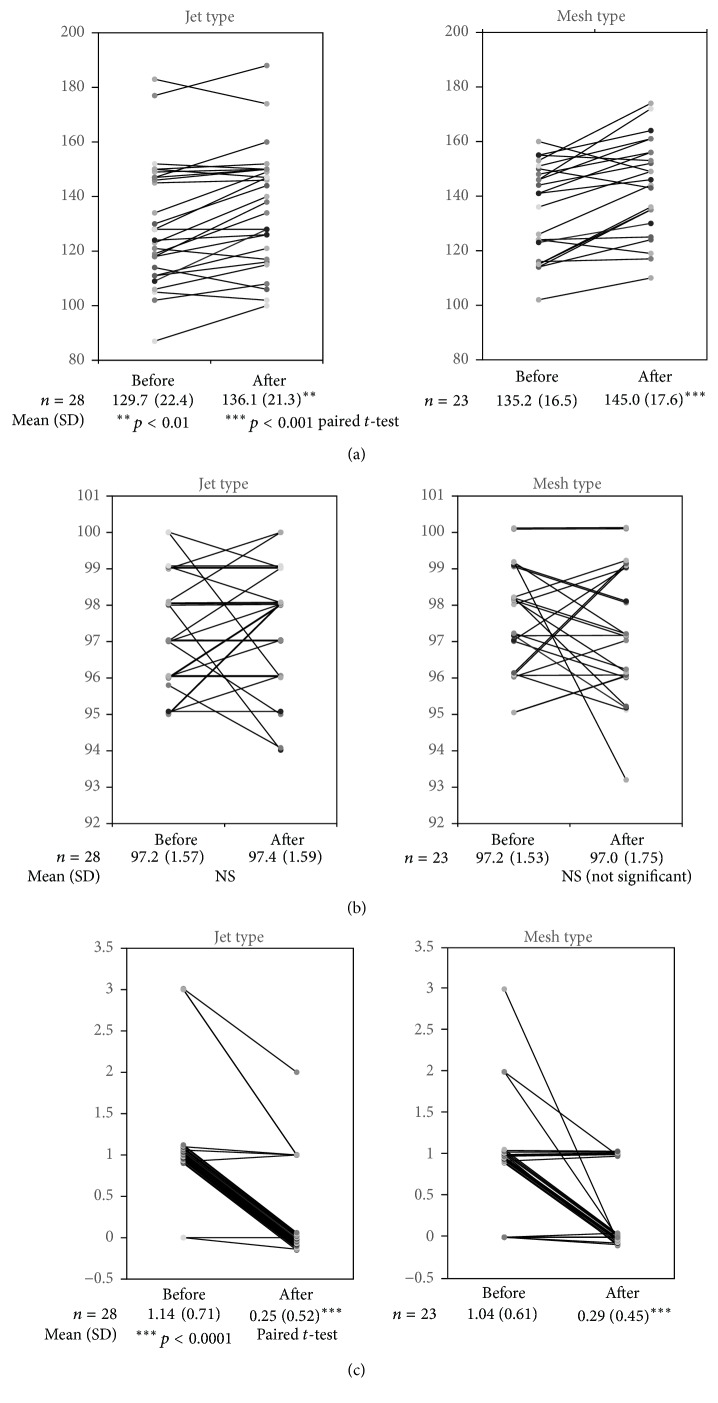
Changes in children ≥ 2 years old. (a) Changes in heart rate. (b) Changes in SpO2. (c) Changes in symptom score.

**Figure 2:**
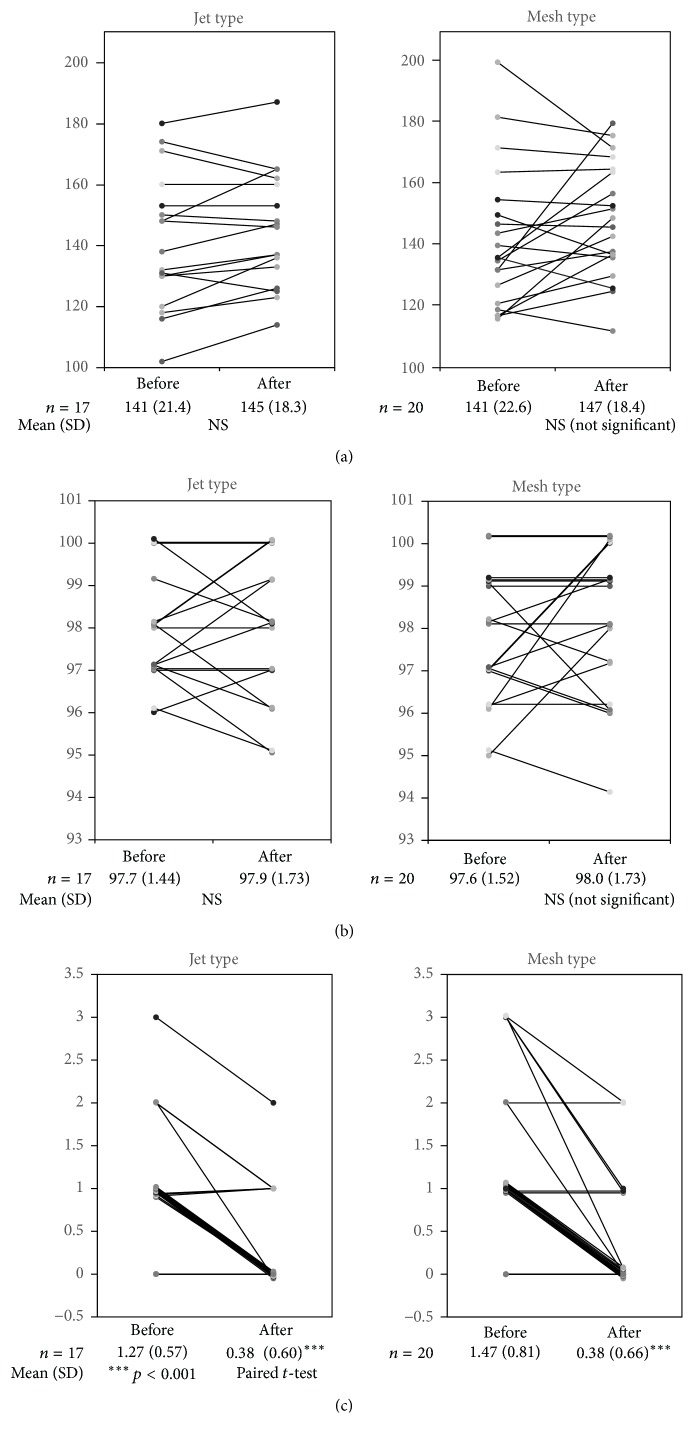
Changes in children < 2 years old. (a) Changes in heart rate. (b) Changes in SpO2. (c) Changes in symptom score.

The mean heart rate in the jet group increased significantly from 129 beats/min to 136 beats/min in patients ≧ 2 years old (*p* < 0.001, paired *t*-test) (Figure 1(a)), but no meaningful change was seen in patients < 2 years old (mean heart rate, from 141 beats/min to 145 beats/min) (Figure 2(a)). In the mesh group, the mean heart rate also increased significantly from 135 beats/min to 145 beats/min among patients ≧ 2 years old (*p* < 0.0001, paired *t*-test) (Figure 1(a)), but no meaningful change was seen in patients < 2 years old (Figure 2(a)). SpO2 before and after inhalation SpO2 was >95% in almost all patients before inhalation therapy was initiated. No significant changes were seen before and after inhalation therapy, and because SpO2 remained within the normal limits before inhalation therapy, improvement effects were difficult to detect (Figures 1(b) and 2(b)).

### 3.3. Clinical Scores before and after Inhalation

The mean clinical symptom score in the jet group decreased significantly from 1.14 before inhalation therapy to 0.25 after in patients ≧ 2 years old (*p* < 0.0001, paired *t*-test) (Figure 1(c)), and from 1.27 to 0.38 in patients < 2 years old (*p* < 0.0001, paired *t*-test) (Figure 2(c)). Likewise, in the mesh group, the mean clinical symptom score decreased significantly from 1.04 to 0.29 in patients ≧ 2 years old (*p* < 0.0001, paired *t*-test) (Figure 1(c)), and from 1.47 to 0.38 in patients < 2 years old (*p* < 0.0001, paired *t*-test) (Figure 2(c)).

## 4. Discussion

To our knowledge, this is the first study to investigate the usefulness of mesh nebulizers in children <6 years old with asthma attacks who are unable to adequately perform spirometry. In the mesh nebulizer device, drug particles are propelled through uniformly sized pores in a manner completely different from a conventional jet nebulizer.

The present study compared the clinical efficacy between the eMotion mesh nebulizer (PARI) and a conventional jet nebulizer (Turbo Boy N; PARI). The mesh nebulizer was not superior to the jet nebulizer in terms of asthma symptom improvement in either young (age < 2 years) or older (age of 2–6 years) pediatric populations.

### 4.1. Advantages in the Basic Performance of a Mesh Nebulizer

Mesh nebulizers have higher aerosol output volumes than other nebulizers. This is accomplished by differing mechanisms of aerosol output. The mesh of the eMotion nebulizer vibrates to discharge the aerosol, while the mesh of NE-U22 nebulizer accepts vibrating air flow to discharge the aerosol. These vibrating-mesh nebulizers can discharge large quantities of aerosol per unit of time producing a shortened inhalation time [4]. Whether the shorter time of inhalation translated to improved clinical outcomes was the subject of our study.

### 4.2. Advantages in the Clinical Use of Mesh Nebulizers

Various reports have described the clinical advantages of mesh nebulizers. Ari et al. [5] found that the delivery efficiency of a mesh nebulizer was higher than a jet nebulizer in all conditions tested. Tezuka et al. [6] reported efficacy and safety of budesonide inhalation. With efficacy by assessed symptoms score and safety assessed by plasma cortisol, mesh nebulizer of 0.25 mg budesonide inhalation is effective and safe in young asthmatic children. Other studies have demonstrated [7] the use of a vibrating-mesh membrane nebulizer connected to the anesthesia circuit for treating bronchospasm. Adachi et al. [1] also reported no adverse reactions in children with asthma treated with procaterol at 1 *μ*g/kg body weight via an eMotion mesh nebulizer.

Among three brands of mesh nebulizers, one multicenter clinical study found that the Aeroneb Go with Idehaler had the most rapid output [8]. However, another study comparing the three mesh-type nebulizers using salbutamol as the bronchodilator found no significant functional differences [9]. Hess suggested that both mesh and jet aerosol delivery devices can work equally well for patients [10]. Unfortunately, many patients use these devices incorrectly, so proper patient education is critical for their proper use.

### 4.3. Salbutamol Dosages with Jet and Mesh Nebulizers

In this study, inhaled doses of salbutamol followed our standard outpatient doses of 0.2 ml in children ≧ 2 years old and 0.1 ml in children < 2 years old. An increased heart rate, which is a known adverse reaction to inhaled salbutamol, occurred in both jet and mesh groups for children ≧ 2 years old (*p* < 0.0001, paired *t*-test). Among children < 2 years old, symptom scores decreased significantly after inhalation therapy despite an absence of any significant increase in heart rate. This suggests that the dose of salbutamol inhalation solution may be excessive in children ≧ 2 years old.

One puff of salbutamol via a pMDI represents a dose of 100 microg; the usual dose in adults is 2 puffs (200 microg). In contrast, salbutamol for inhalation is a 0.5% solution with a dose of 500 microg in 0.1 ml or 1000 microg in 0.2 ml. The usual dose of 0.2 ml in children ≧ 2 years old is thus equivalent to ~10 puffs of salbutamol via a pMDI, representing 5 and 10 times the usual adult and child doses, respectively. The package insert for salbutamol inhalation solution recommends a dose of 0.3–0.5 ml in adults and 0.1–0.3 ml in children. Moreover, because the inhalation efficiency of electronic nebulizers has also improved with the move from ultrasonic to jet or mesh devices and from persistent inhalation to intermittent inhalation, the doses currently used for adults and children should be reconsidered. We considered that, for children ≧ 2 years, a 0.2 ml salbutamol solution is an excessive dose for a mild asthma attack. But for moderate to severe attacks, this dose may be adequate due to air flow limitation preventing the drug from reaching obstructed sites.

## 5. Limitations

Although this study included all patients attending Murayama Pediatrics, asthma severities in this study were mild. Future studies should test patients with moderate or severe airflow obstruction since there could be differences in clinical outcomes if treatment time is more clinically important. Thus, it is difficult to examine adequate salbutamol inhalation doses for moderate and severe attacks with data from this study.

## 6. Conclusion

For children with mild asthma, there was no significant difference between clinical scores after beta 2 inhalation therapies with either PARI Turbo Boy N (jet type) or eMotion (mesh type). However, there was a significant difference in required inhalation time, with eMotion nebulizer demonstrating a shortened time. Children ≧ 2 years with mild asthma attacks experienced a significantly increased heart rate in both mesh and jet nebulizer treatment groups. The dose of salbutamol (0.2 ml for ≧2 years) used for asthma attacks should be reconsidered for mild asthma.

## Figures and Tables

**Table 1:** Clinical examinations before and after treatment for children ≥ 2 years old.

>2 years	Jet type		Mesh type	
	Before	After	Before	After
*n*	28		23	
Age, years	3.55 (1.17)		3.44 (0.90)	
Inhalation time, s	377 (58.7)		248 (31.4)^*∗∗∗*^	
Heart rate, beats/min	129 (22.4)	136 (21.3)^*∗∗*^	135 (16.5)	145 (17.6)^*∗∗∗*^
SpO_2_, %	97.2 (1.57)	97.4 (1.59)	97.2 (1.53)	97.0 (1.59)
Symptom score	1.14 (0.71)	0.25 (0.52)^*∗∗∗*^	1.04 (0.61)	0.29 (0.45)^*∗∗∗*^

Mean (SD), ^*∗∗*^*p* < 0.001, ^*∗∗∗*^*p* < 0.0001, paired *t*-test, and independent *t*-test.

**Table 2:** Clinical examinations before and after treatment for children < 2 years old.

<2 years	Jet type		Mesh type	
	Before	After	Before	After
*n*	17		20	
Age, years	1.30 (0.35)		1.38 (0.40)	
Inhalation time, s	364 (53.9)		236 (22.5)^*∗∗∗*^	
Heart rate, beats/min	141 (21.4)	145 (18.3)	141 (22.6)	147 (18.4)
SaO_2_, %	97.7 (1.44)	97.9 (1.73)	97.6 (1.52)	98.0 (1.73)
Symptom score	1.27 (0.57)	0.38 (0.60)^*∗∗∗*^	1.47 (0.81)	0.38 (0.66)^*∗∗∗*^

Mean (SD), ^*∗∗∗*^*p* < 0.0001, paired *t*-test, and independent *t*-test.

## Abbreviations

pMDI: Pressurized Metered-Dose Inhalation
SpO2: Peripheral oxygen saturation.
